# Combined STN/SNr-DBS for the treatment of refractory gait disturbances in Parkinson's disease: study protocol for a randomized controlled trial

**DOI:** 10.1186/1745-6215-12-222

**Published:** 2011-10-11

**Authors:** Daniel Weiss, Tobias Wächter, Christoph Meisner, Melanie Fritz, Alireza Gharabaghi, Christian Plewnia, Sorin Breit, Rejko Krüger

**Affiliations:** 1 German Centre of Neurodegenerative Diseases, Tübingen, Germany; 2 Department for Neurodegenerative Diseases and Hertie Institute for Clinical Brain Research, University of Tübingen, Germany; 3 Department of Medical Biometry, University of Tübingen, Tübingen, Germany; 4 Werner Reichardt Centre for Integrative Neuroscience and Department of Neurosurgery, University of Tübingen, Germany; 5 Department of Psychiatry, University of Tübingen, Tübingen, Germany

**Keywords:** balance, deep brain stimulation (DBS), freezing of gait (FOG), gait disturbance, interleaved pulses, Parkinson's disease, stimulation, substantia nigra pars reticulata (SNr), subthalamic nucleus (STN)

## Abstract

**Background:**

Severe gait disturbances in idiopathic Parkinson's disease (PD) are observed in up to 80% of all patients in advanced disease stages with important impact on quality of life. There is an unmet need for further symptomatic therapeutic strategies, particularly as gait disturbances generally respond unfavourably to dopaminergic medication and conventional deep brain stimulation of the subthalamic nucleus in advanced disease stages. Recent pathophysiological research pointed to nigro-pontine networks entrained to locomotor integration. Stimulation of the pedunculopontine nucleus is currently under investigation, however, hitherto remains controversial. The substantia nigra pars reticulata (SNr) - entrained into integrative locomotor networks - is pathologically overactive in PD. High-frequent stimulation of the substantia nigra pars reticulata preferentially modulated axial symptoms and therefore is suggested as a novel therapeutic candidate target for neuromodulation of refractory gait disturbances in PD.

**Methods:**

12 patients with idiopathic Parkinson's disease and refractory gait disturbances under best individual subthalamic nucleus stimulation and dopaminergic medication will be enroled into this double-blind 2 × 2 cross-over clinical trial. The treatment consists of two different stimulation settings using *(i) *conventional stimulation of the subthalamic nucleus [STNmono] and *(ii) *combined stimulation of distant electrode contacts located in the subthalamic nucleus and caudal border zone of STN and substantia nigra pars reticulata [STN+SNr]. The primary outcome measure is the change of the cumulative 'axial score' (UPDRS II items '13-15' and UPRDS III items '27-31') at three weeks of constant stimulation in either condition. Secondary outcome measures include specific scores on freezing of gait, balance function, quality of life, non-motor symptoms, and neuropsychiatric symptoms. The aim of the present trial is to investigate the efficacy and safety of a three week constant combined stimulation on [STN+SNr] compared to [STNmono]. The results will clarify, whether stimulation on nigral contacts additional to subthalamic stimulation will improve therapeutic response of otherwise refractory gait disturbances in PD.

**Trial registration:**

The trial was registered with the clinical trials register of http://www.clinicaltrials.gov (NCT01355835)

## Background

### Refractory gait disturbances in Parkinson's disease

Severe gait disturbances in idiopathic Parkinson's disease are observed in up to 80% of the patients in advanced disease stages [1,2] with important impact on quality of life [3-5]. Therapeutic deep brain stimulation of the subthalamic nucleus (STN-DBS) as an evidence-based therapy [6-8] generally ameliorates segmental symptoms and motor fluctuations, whereas axial symptoms and in particular gait disturbances may respond unfavourably and generally aggravate in parallel with the underlying neurodegeneration [9,3,10]. In this condition, increasing intensity of high-frequent STN-DBS at 130 Hz even worsens the condition [10]. Currently, several approaches are under investigation in order to address the therapeutic need for gait disturbances refractory to dopaminergic treatment and STN-DBS. STN-DBS on lower frequencies, e.g. at 60 Hz can improve gait disturbances, however is limited by the recurrence of segmental symptoms like tremor, bradykinesia and rigidity [10]. Stimulation of the pedunculopontine area for refractory gait disturbances remains controversial at the moment [11-13], however several experimental lines of evidence demonstrated the integrative role of reciprocal brainstem circuitries including substantia nigra pars reticulata (SNr) and the pedunculopontine area [14-16]. Importantly, activity of the SNr can be modulated after implantation for conventional STN-DBS, as the caudal electrode contacts are generally located in the caudal border zone of STN and SNr [17,18].

### Simultaneous stimulation of STN and SNr with *interleaved pulses*: uncovering of a novel stimulation paradigm for refractory gait disturbances?

At present, stimulation impulses are generally delivered on single monopolar contacts polarised against the generator case or between bipolar adjacent electrode contacts. Recently, the advancement of the implantable impulse generators enabled the novel paradigm of the so-called *'interleaved pulses'*, i.e. stimulation impulses are delivered simultaneously on two different contacts in alternating order. Importantly, each of the contacts can be programmed onto specific parameters (amplitude, pulse width) at a common stimulation frequency (e.g. 125 Hz on each contact). This might allow for *(i) *optimization of the stimulation settings by minimizing side effects from current spreading to the vicinity of the STN (especially if stimulation on a second monopolar contact is necessary to achieve sufficient therapeutic effects without inducing side effects), and *(ii) *improved therapeutic efficacy by delivering the stimulation pulses more selectively on different and distant contacts, although both issues remain to be demonstrated in clinical studies.

In our center for deep brain stimulation electrodes in STN-DBS surgery are generally placed with the uppermost contact of the quadripolar electrode located in the rostral border zone of zona incerta and STN and the lowermost contact in the caudal border zone of STN and SNr. This neuroanatomic assumption is substantiated by several common intra- and postoperative findings: *(i) *efficacy of a proximal contact on segmental symptoms like rigidity or tremor and a facultative induction of dyskinesias on the respective contact (upon increasing energy delivery) indicates its localisation in the dorsolateral STN motor area; *(ii) *intraoperative single cell recordings often identify bursting STN activity followed by regular tonic discharge when the descending microelectrode is leaving STN and entering SNr [19]. The novel paradigm using *"interleaved pulses" *therefore enables simultaneous stimulation with specific parameters of both STN area and the caudal STN/SNr border zone.

### Objectives

We hypothesize that simultaneous stimulation on contacts located in both STN and caudal STN/SNr border zone [STN+SNr-DBS] improves gait disturbances compared to conventional STN-DBS delivered on a single contact located in the STN area [STNmono].

➢ To test the hypothesis that [STN+SNr-DBS] is more effective on hypokinetic gait disturbances compared to [STNmono] in a randomised double-blind cross-over three week follow-up.

➢ To evaluate the short term effects of the conditions [StimOff], [STNmono] and [STN+SNr] on hypokinetic gait disturbances.

➢ To assess the impact of combined [STN+SNr-DBS] on further specific gait and balance features including freezing of gait in particular, as well as further non-motor features of Parkinson's disease in terms of quality of life, non-motor and neuropsychiatric symptoms as secondary endpoints.

## Design and Methods

This double-blind randomised cross-over clinical trial consists of two arms. Severity of the gait disturbance will be assessed after a three week follow-up of both *(i) *conventional stimulation of the STN area [STNmono] and *(ii) *combined stimulation of the STN area and the caudal STN/SNr border zone [STN+SNr]. 12 consecutive patients will be randomised on blocks to the two treatment conditions in a 1:1 ratio and treatments will be crossed-over after three weeks of follow-up, respectively. Moreover, a baseline assessment *'off dopaminergic medication' *will be performed in order to assess short-term effects and to assure optimal stimulation parameters of the conventional STN-DBS [STNmono]. A composite 'axial score' including the major clinical and anamnestic items on gait, posture and balance function from UPDRSII (items 13-15) and UPDRS III (items 27-31) constitutes the primary outcome measure.

### Population and Recruitment

The patient population will consist of patients with refractory gait disturbances and advanced Parkinson's disease treated with STN-DBS with implanted impulse generators that allow for the delivery of *"interleaved pulses" *(ACTIVA PC, ACTIVA RC). Patients will be recruited at the Center of Neurology, Tübingen University.

### Inclusion criteria

• Written informed consent

• Age: between 18 and 80 years

• Idiopathic Parkinson's disease (according to the "British Brain Bank criteria" [20] including genetic forms and therapy with STN-DBS (ACTIVA pulse generator)

• Optimized subthalamic stimulation (refer 'treatment' section)

• Gait disturbance refractory to best individual STN-DBS (STNmono) and dopaminergic therapy: composite **'axial score' **in the best clinical [MedOn/STNmono] condition ≥ 12

• Clinical and image-guided or electrophysiological confirmation of *(i) *at least one of the two rostral contacts of the quadripolar electrode localized in the STN area.

• Dopaminergic medication constant for at least four weeks prior to study enrolment

• Implantation of the DBS electrodes at least 6 months before study enrolment

• Disease duration ≥ 5 years

### Exclusion criteria

• Cognitive impairment (Mini Mental State Exam < 25)

• Participation in other clinical trials within the past three months and during enrolment in our study

• Suicidality, Psychosis

• Other severe pathological chronic condition that might confound treatment effects or interpretation of the data

• Pregnancy

• Acute adverse effects from stimulation on contacts in the caudal STN/SNr border zone

#### Screening

Patients will be recruited at the Department for Neurodegenerative Diseases of the Center of Neurology, University of Tübingen, Germany. A neurologist will assess the eligibility of a patient according to the inclusion and exclusion criteria as detailed above.

#### Safety

##### Endpoints of safety are

• Death

• Severe symptomatic exacerbation of the pre-existing gait disturbance, defined by repetive falls (if not preexistant) due to aggravated freezing of gait or imbalance (UPDRS II, items 13-14, UPDRS III, item 30)

• Newly occurring or aggravated depressive symptoms (BDI), suicidality (BDI, item 9), impulsivity (Barrett impusivity scale), hallucinatory behaviour and psychosis (UPDRS I, item 2).

• worsening of segmental motor symptoms (UPDRS III, items 20-26) or motor fluctuations (UPDRS IV)

Information about these safety parameters is recorded in the CRF.

### Outcome measures

The primary outcome measure is defined as the difference of the composite 'axial score' including the major UPDRS II and III items of gait, balance and posture after three weeks of double-blind treatment with either *(i) *conventional [STNmono] or *(ii) *combined [STN+SNr-DBS]. The secondary efficacy variables enable a differentiated assessment of specified axial symptoms, namely freezing of gait (including provoking manoeuvres), gait velocity, clinical balance testing, neuropsychiatric symptoms, and non-motor symptoms.

### Study protocol

Clinical testings at baseline will be performed after overnight withdrawal of dopaminergic medication and after optimization of subthalamic stimulation. Follow-up examinations will be performed in the *'dopaminergic on state'*. Of note, no follow-up examinations with a single active SNr contact will be conducted as this previously failed to control for segmental symptoms like tremor, bradykinesia and rigidity [17].

**Figure 1:**
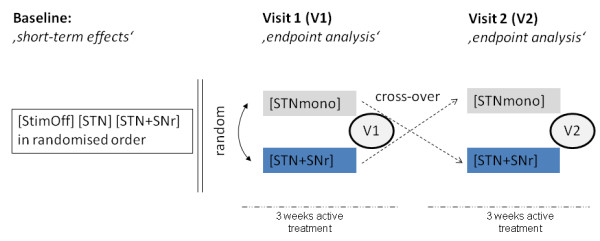
**Study design**.

Figure 1: Baseline testings will be performed *"off dopaminergic medication" *after overnight withdrawal with *(i) *[StimOff], *(ii) *[STNmono], and *(iii) *[STN+SNr]. On visits 1 and 2, we will assess the treatment effects of [STNmono] and [STN+SNr] after three weeks of constant stimulation on either setting. The order of the treatment conditions will be randomized and crossed after the first visit. Randomisation lists for the different treatment conditions are prepared by the Department of Medical Biometry.

### Withdrawal from the study

In case of endangerment of personal safety or lack of compliance or withdrawal of informed consent, a patient will instantly be excluded from further participation in the study.

### Sample size calculation and statistical analysis plan

#### Primary Endpoint

The primary endpoint for the statistical evaluation of the therapy is the change in composite 'axial score'. The 'axial score' is built by 8 items from the UPDRS II and III, all 5-point rated. For the statistical evaluation the five rating points are represented by the numbers 0 to 4, which represent increasing levels of pathology. The 'axial score' will be scored by the sum of the ratings across the 8 items (range 0 to 32). As change in UPDRS scores is a common primary efficacy outcome measure in Parkinson's disease and only items of the original UPDRS are required for the definition of the primary endpoint the statistical evaluation methods should be based on the psychometric validation of the UPDRS and no own validation studies are necessary.

The primary endpoint for the statistical evaluation will be the change in 'axial score' from baseline to visit 2 (after 6 weeks). For every patient we will determine two change-scores for the two phases in the Cross-Over. By means of a paired t-test the null hypothesis of equality of the two therapies concerning the change in 'axial score' will be tested. The decision for maintaining or rejecting the null hypothesis will be made applying a two-sided test with α = 0.05. The observed effects will be described by use of means and effect sizes including the appropriate 95%-confidence intervals. The confirmatory statistical evaluation of the efficacy of the [STN+SNr-DBS] in this trial will be restricted to the primary endpoint. Only the rejection of the null hypothesis will be interpreted as statistical evidence for the efficacy of [STN+SNr-DBS].

As no comparable study is available at the moment, we defined an improvement of 4 points on the primary outcome measure 'axial score' to be clinically relevant on hypokinetic gait disturbances and assumed a standard deviation of 4.0 (effect size: 1.0). A sample size of 10 will have 80% power to detect a difference in means of 4.0 (e.g. first condition mean: 16.0 and a second condition mean of 12.0), using a paired t-test with a 0.05 two-sided significance level (sample size estimated using NQuery Advisor 7.0). To adjust for a maximum of two dropouts a total of n = 12 patients will be included in the study.

#### Secondary Endpoints

As all secondary endpoints are based on validated scores, we assume that parametric statistical methods can be used for the analysis. The secondary endpoints will be compared and statistically assessed for descriptive purposes and not in a confirmatory sense. The aim is explorative data analysis, not hypothesis testing or generation of evidence for efficacy. Because of the explorative character of this part of the analysis, no a priori statistical analysis plan exists. If adequate, changes of scores over time will be analysed with paired t-tests or appropriate statistical methods (eg. Repeated Measurements Anova). If a categorization of scores should be adequate (eg. classification in success vs. failure according to score-cut-offs) we will use adequate analysis methods for categorical variables (eg. McNemar Test). In addition, appropriate statistical methods of explorative data analysis including graphical methods and descriptive statistics will be used. No interim analysis and no subgroup analysis are planned.

#### Handling of Missing Data

All variables included in the CRF are mandatory. The monitoring will assure quality of the assessments. Thus, missing values are to be expected (eg. refusal of patients). Patients with missing values for the primary endpoint will be included with the last observed score before the planned time point (last-observation-carried-forward). All randomised patients will be included (Intention-to-treat).

### Randomisation

A randomisation list was prepared by the Department of Medical Biometry of the University Hospital of Tübingen.

### Treatment

Optimization of subthalamic stimulation is mandatory prior to study enrolment. Based on the current knowledge for optimized DBS programming *(i) *gait disturbances emerge and progress in the first years after introduction of subthalamic stimulation [9], *(ii) a *further increase of the stimulation amplitudes may even aggravate the gait disturbance [10], and *(iii) *the stimulation intensity of the lower extremity with longer step length should be reduced compared to the worse affected side - this was previously demonstrated to ameliorate gait symmetry with benefitial effects to gait disturbances, presumably freezing of gait [21]. These actions will be taken before considering combined [STN+SNr] stimulation, however, even if applied a substantial proportion of patients continues to exhibit refractory gait disturbances.

After written informed consent and screening for inclusion criteria, patients will be examined in the baseline condition *‚off dopaminergic medications' *in order to assure optimization of the best individual stimulation parameters of the conventional STN stimulation [STNmono] and to determine the short-term effects of either stimulation setting. The [STN+SNr] condition will consist of the [STNmono] parameters and additional simultaneous stimulation with *interleaveing pulses *on a distal contact with best individual amplitude, 60 μs pulse width, and 125 Hz frequency will be introduced depending on individual thresholds for side effects from current spreading. In the baseline examination a randomised clinical evaluation of the treatment conditions [StimOff], [STNmono], and [STN+SNr] in terms of short term effects will be performed.

After the baseline examination, patients will be randomised to either [STNmono] or [STN+SNr] treatment and scheduled at three weeks of constant stimulation (Visit 1). After this first endpoint assessment treatment will be crossed-over and patients will be re-scheduled after further three weeks of constant stimulation for the second endpoint assessment (Visit 2). The follow-up period is - due to the current clinical evidence - sufficiently long to control adequately for carry-over effects since the endpoint assessment in the follow-up period will be scheduled only after three weeks of constant stimulation on either setting. STN-DBS in PD generally evokes clinical effects within short time intervals ranging from several seconds to few hours and presents completely reversible in the same time range. In the first patient described with improvement from [STN+SNr] compared to [STNmono], the clinical superiority of [STN+SNr] was demonstrated after 30 minutes of constant stimulation on either setting [18], and monopolar stimulation on STN or SNr contacts revealed discriminable clinical effects on segmental and axial symptoms if both settings were applied randomly within one day in an independent study [17]. Dopaminergic medication will be held constant during the whole six week cross-over period and should be stable for at least 4 weeks before study enrolment.

Importantly, we did not consider stimulation on a single monopolar contact in the SNr area in our study design as this failed to improve segmental symptoms previously [17], and therefore is not applicable for adequate management of Parkinsonian motor symptoms.

### Blinding

The endpoint assessor remains masked to the treatment until the final data analysis. In order to limit potential patients' knowledge on the stimulation parameters, stimulation parameters will be strictly kept subthreshold for side-effects. The parameters will be changed several times between [STNmono] and [STN+SNr] before the parameters of interest are maintained. Patients and endpoint assessor will be blinded to both short-term testings and follow-up visits, so that a possible clinical improvement of gait on either setting will remain unattributable for both patient and endpoint assessor.

### Ethics, Consent, Study Organization and Registration

The trial will be conducted in agreement with the principles of the Declaration of Helsinki, and with the guidelines of Good Clinical Practice (GCP) of the International Conference on Harmonisation of Technical Requirements for Registration of Pharmaceuticals for Human Use (ICH). The study protocol was approved by the local and independent Ethics Committee Tübingen (Institutional Review Board). Extramural funding for the present study is provided by Medtronic GmbH (Medtronic, Meerbusch, Germany). The investigator will explain the benefits and risks of participation in the study to each subject and will provide an informed consent form approved by the independent ethics committee. Only patients, who sign the form, will be included in the study. Results will be published anonymously. If there is an unexpected worsening of motor or neuropsychiatric symptoms, patients can immediately contact our clinic 24 hours a day and will be immediately referred to a neurologist specialised on deep brain stimulation.

### Data Management

Case report forms must be completed according to the following schedule:

a) Before the treatment starts: the patient must be screened/randomised. For that purpose all relevant data must be reported.

b) Documentation of the treatment and follow-up visits: Each visit should be documented immediately.

c) Upon occurrence of a Severe Adverse Event (SAE)

All SAEs occurring during the observation period of 9 months must be reported by fax to the sponsor's medical expert, the medical director of the Department of Neurodegenerative Diseases of the Center of Neurology, Tuebingen University. All forms must be dated and signed by the responsible investigator or one of his/her authorized staff members.

All data will be documented on CRFs by authorized investigators and monitored for completeness and correctness. Only complete and correct data will be entered into the study data base. The study software *koordobas*, an Oracle-based application of the department of Medical Biometry, will be used for the data management.

In all cases, it remains the responsibility of the investigator to check that case report forms are completely and correctly filled in. The data manager will perform extensive consistency checks on the CRFs and issue Query Forms in case of inconsistent data. Those Query Forms must be immediately answered and signed by the investigator (or an authorized staff member). The original must be returned to the data manager and a copy must be appended to the investigator's copy of the CRFs.

All study related data (electronic as well as on paper) will be stored for 10 years in the archive of the Department of Neurodegenerative Diseases of the Center of Neurology, Tübingen University, University of Tübingen.

Assessment, storing, processing, and deleting of person related data will be conducted in accordance to German law.

## Discussion

The treatment of hypokinetic gait disturbances refractory to dopaminergic medication and conventional STN-DBS constitutes an unmet therapeutic need with important impact on quality of life. Current concepts aim on neuromodulation of reciprocal functional locomotor circuitries of basal ganglia and brainstem that hold an integrative key role within the locomotor system. This double-blind controlled cross-over phase II clinical trial has been designed to evaluate both short-term and long-term efficacy of a novel stimulation paradigm using '*interleaved pulses' *on subthalamic and nigral electrode contacts for refractory gait disturbances. Based on the effect size and standard deviation of the Intention-to-treat analysis a larger clinical efficacy study will be designed.

## Abbreviations

DBS: deep brain stimulation; PD: Parkinson's disease; STN: subthalamic nucleus; SNr: substantia nigra pars reticulate; [STNmono]: conventional stimulation on subthalamic electrode contacts; [STN+SNr]: combined stimulation on electrode contacts located in the subthalamic nucleus and substantia nigra pars reticulata with interleaved pulses.

## Competing interests

The study is funded by Medtronic GmbH (Medtronic, Meerbusch, Germany).

Daniel Weiss receives a research grant from the University of Tübingen [AKF 259-0-0] and a research grant from Medtronic. Daniel Weiss received speaker's honoria and travel grants from Medtronic, Solvay Pharmaceuticals, UCB, and the Movement Disorders Society

Tobias Wächter has received speaker's honoraria and travel grants from Medtronic, Schwarz Pharma, and Solvay.

Christoph Meisner reports no disclosures.

Melanie Fritz reports no disclosures.

Alireza Gharabaghi is supported by grants from the German Research Council [DFG GH 94/2-1, DFG EC 307], Federal Ministry for Education and Research [Bernstein 01GQ0761, BMBF 16SV3783], Medtronic Research Grant, and European Union [ERC 2276329] and from the University of Tübingen [AKF 238-0-0].

Christian Plewnia received research grants from the German Research Council [DFG, 253 PL1-1] and University of Tübingen [AKF 238-0-0].

Sorin Breit receives a research grant from University of Tübingen [AKF 246-0-0].

Rejko Krüger R. is supported by grants of the German Research Council [DFG; KR2119/3-2, the Michael J Fox Foundation, the Federal Ministry for Education and Research [BMBF, NGFNplus; 01GS08134] and from the Medical Faculty of the University of Tübingen [AKF 238-0-0].

## Authors' contributions

All authors provided substantive intellectual contributions to the study design. DW is the initiator of the study; DW and RK are principal investigators of the study. CM is the trial statistician and carried out the randomisation and sample size calculations. DW is the trial manager. All authors drafted the original study protocol. The first draft of the manuscript was written by DW, CM, and RK. All authors have read and reviewed critically the manuscript for intellectual content and approved the final version of the manuscript.
